# Decreasing incidence of complex regional pain syndrome in the Netherlands: a retrospective multicenter study

**DOI:** 10.1177/20494637211041935

**Published:** 2021-09-06

**Authors:** Tjitske D Groenveld, Emily Z Boersma, Taco J Blokhuis, Frank W Bloemers, Jan Paul M Frölke

**Affiliations:** 1 Department of Surgery, Radboud University Medical Center, Nijmegen, The Netherlands; 2 Department of Surgery, Maastricht University Medical Center, Maastricht, The Netherlands; 3 Department of Surgery, VU University Medical Center, Amsterdam, The Netherlands

**Keywords:** Complex regional pain syndrome, posttraumatic dystrophy, Sudeck’s dystrophy, distal radius fracture, incidence

## Abstract

**Background:**

Complex regional pain syndrome type I (CRPS) is a symptom-based diagnosis of which the reported incidence varies widely. In daily practice, there appears to be a decrease in incidence of CRPS after a distal radius fracture and in general.

**Questions/purposes:**

The aim of this study was to assess the trend in the incidence of CRPS after a distal radius fracture and in general in the Netherlands from 2014 to 2018.

**Methods:**

The incidence of CRPS after a distal radius fracture was calculated by dividing the number of confirmed cases of CRPS after distal radius fracture by the total number of patients diagnosed with a distal radius fracture. Medical records of these patients were reviewed. Hospital-based data were used to establish a trend in incidence of CRPS in general. A Dutch national database was used to measure the trend in the incidence of CRPS in the Netherlands by calculating annual incidence rates: the number of new CRPS cases, collected from the national database, divided by the Dutch mid-year population.

**Results:**

The incidence of CRPS after distal radius fracture over the whole study period was 0.36%. Hospital data showed an absolute decrease in CRPS cases from 520 in 2014 to 223 in 2018. National data confirmed this with a decrease in annual incidence from 23.2 (95% CI: 22.5–23.9) per 100,000 person years in 2014 to 16.1 (95% CI: 15.5–16.7) per 100,000 person years in 2018.

**Conclusion:**

A decreasing trend of CRPS is shown in this study. We hypothesize this to be the result of the changing approach towards CRPS and fracture management, with more focus on prevention and the psychological aspects of disproportionate posttraumatic pain.

**Level of Evidence:**

level 3 (retrospective cohort study).

## Introduction

Complex regional pain syndrome type I (CRPS), also known as posttraumatic dystrophy or Sudeck’s dystrophy, is an unexplained pain syndrome that can occur following a variety of events. CRPS most commonly occurs after a fracture.^1,2^ It is characterized by disproportionate pain and accompanying autonomic and motor disturbances.^3,4^ The reported incidence of CRPS varies widely between 5.5 per 100,000 person-years in the United States and 26.2 per 100,000 person-years in the Netherlands.^1,2^ CRPS, specifically after a distal radius fracture (DRF), is frequently studied and has a reported incidence between 0.8% and 37%.^5–8^

Over the years, many suspected pathophysiological causes have been proposed, but scientific evidence is scarce and of low quality.^9,10^ Consequently, a gold standard for the diagnosis of CRPS could never be established. Currently, the Budapest criteria are the best validated and most common internationally used criteria.^11^ The subjectivity of these criteria has raised the question of whether CRPS is actually a disease on its own or more of a functional pain syndrome.^4,9,12,13^ These doubts are supported by the remarkable similarity between these symptoms and symptoms due to immobilization and disuse.^14–17^ Recently, multiple studies have been performed regarding disuse and CRPS after DRF. Not only have these studies confirmed the similarity between disuse and CRPS in clinical symptoms but they have also identified similar processes in the central nervous system.^18–20^ Based on these recent insights, new prevention and treatment methods have been developed. Recent studies have shown that short immobilization and early exercise after DRF can prevent disuse.^21,22^ Furthermore, graded motor imaging (GMI) and pain exposure physical therapy (PEPT) after DRF, both addressing also the cognitive and behavioural aspects of pain, have shown promising results in recent clinical trials.^23,24^ However, there are no recent data on the incidence of CRPS. In this study, we aimed to determine the 5-year trend in the incidence of CRPS after DRF in the Netherlands from hospital data. Second, we aimed to validate this by estimating the 5-year trend in the incidence of CRPS in the Netherlands from both population-based data and hospital data.

## Methods

### Research design

This study was a retrospective multicenter study. The 5-year trend in the incidence of CRPS was determined using both a national registration of diagnosis treatment combination (DBC) codes and DBC codes from the registries of three trauma centres in the Netherlands (Radboud University Medical Centre, Maastricht University Medical Centre, Amsterdam University Centre (VUmc)) This study was approved by the medical ethics board of our University Hospital (NL2019-5823) as well as the medical ethical boards of the other participating centres (METC 2019-1387, VUmc 2019-3956).

### Case definition and data accumulation

In the Netherlands, all patients seen and treated in a hospital (outpatient and inpatient) are registered using a DBC code. Every specialism has its own range of DBC codes. As a result, the same condition will be scored under a different DBC code when treated by a different specialist. Hospitals are reimbursed based on these DBC codes. These registrations are gathered in a national database, the DBC Information System (DIS). For this study, a combination of open (i.e. freely accessible) DIS data from the national database and data derived from the DBC registration of three trauma centres were used. The following DBC codes corresponding to CRPS were obtained by assessing the different clinical pathways in the participating centres and specialties involved: DBC 150 (anesthesiology), 296 (surgery), and 2110 (orthopaedics). A newly opened DBC code for CRPS was interpreted as a suspected case of CRPS, since they are used for validated diagnoses as well as for patients who are seen by a specialist but who are eventually not diagnosed with CRPS. DBC 212 (surgery) and 3110 (orthopaedics) were identified to correspond with a DRF.

For the calculation of the incidence of CRPS after DRF and for the estimation of the incidence of CRPS in general (based on hospital data), data from the DBC registration of the three participating trauma centres were used.

Two lists of patients were extracted from the hospital registrations: all persons of all ages for whom DBC 212 or 3110 was opened between January 2014 and December 2018 and all persons of all ages for whom DBC 296, 150, or 2110 was opened between January 2014 and June 2019. After extraction, every patient was assigned a unique research ID to ensure confidentiality. By comparing both lists, a selection of patients with the possible diagnosis of CRPS after a DRF was identified. For these patients, the diagnosis was validated by the treating physician by reviewing their medical records. In all hospitals, the same criteria are used for diagnosing CRPS: the Budapest criteria. These criteria have not been changed over the study period. A diagnosis was considered confirmed when the Budapest criteria were fulfilled and the precipitating event was a DRF. Age and sex were extracted from the database to allow for subgroup analyses as well as assessment of the association of age and sex with the incidence of CRPS.

**Figure 1.**
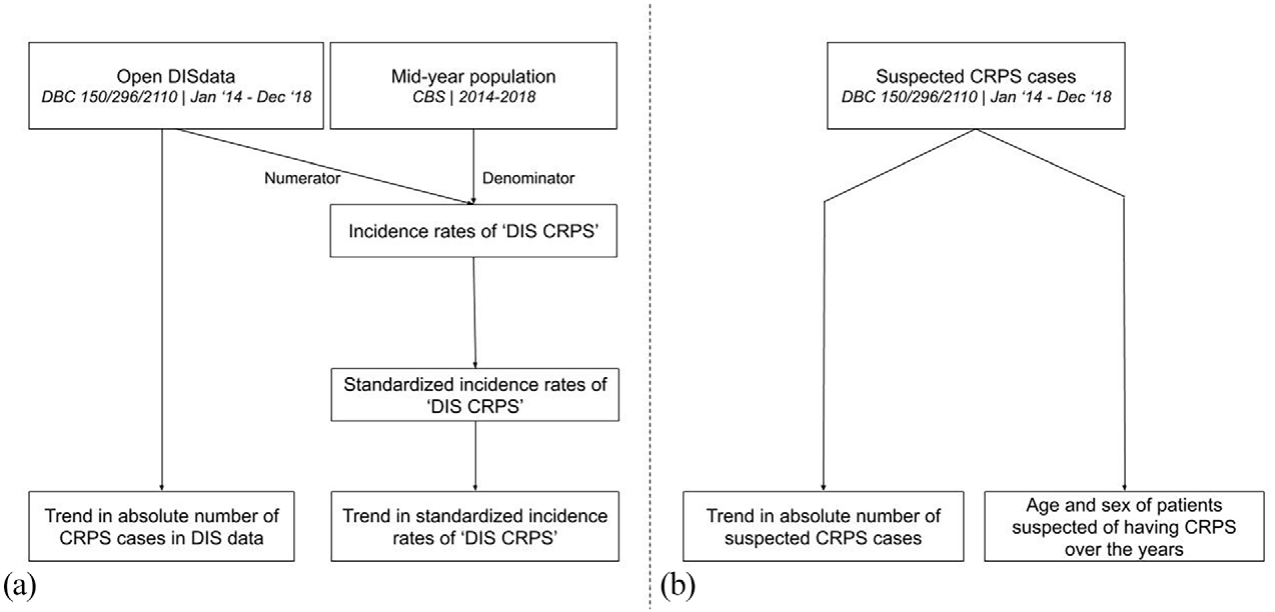
The flowchart shows the process of data accumulation for and analysis of the trend in incidence of suspected CRPS in the Netherlands using (a) population-based data and (b) hospital-based data. DIS CRPS: cases of suspected CRPS derived from DIS data.

**Figure 2.**
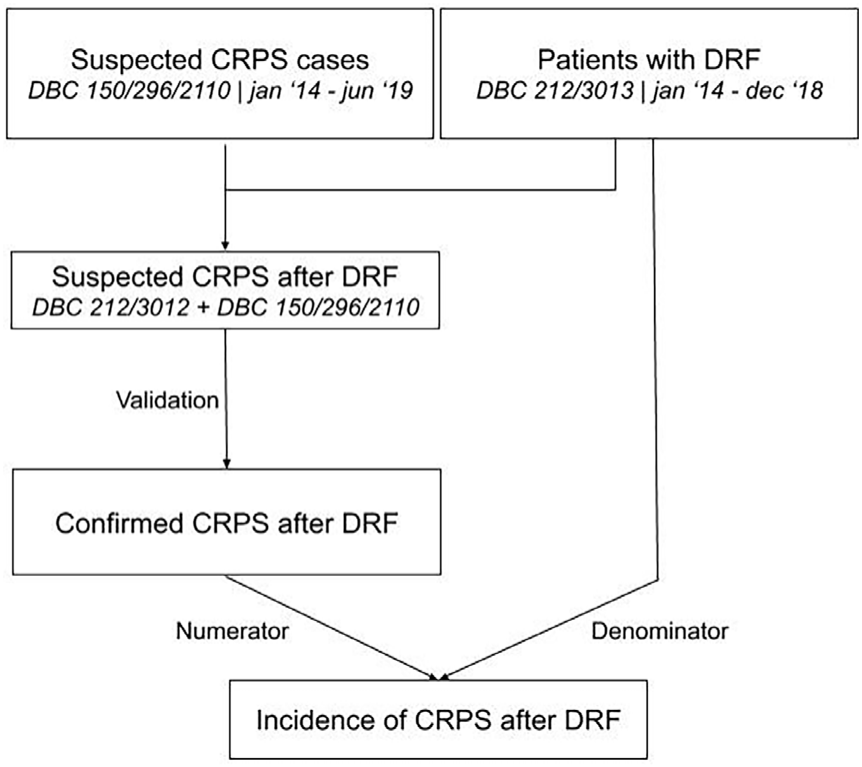
The flowchart shows the process of data accumulation for and analysis of the trend in incidence of CRPS after DRF in the Netherlands.

To determine the trend in the incidence of CRPS in general in the Netherlands, the number of newly opened DBC codes for CRPS for the years 2014 to 2018 was collected using open DIS data from the national database. Data on the mid-year population of the Netherlands were obtained from the Central Bureau for Statistics. For clarification of the process of data accumulation, see Figures 1 and 2.

### Analysis

Data management and analysis were performed using Stata. Standard descriptive statistics were used to compare categorical variables (chi-square test) or means (Student’s *t*-test).

First, the incidence of CRPS after a DRF was calculated by counting the number of confirmed cases of CRPS after a DRF (numerator) and dividing this by the total number of patients diagnosed with a DRF (denominator). Likewise, this ratio was calculated for the separate years together with the corresponding 95% confidence intervals. The 95% confidence intervals were calculated using the Score system. We considered there to be a significant trend when there was no overlap in 95% confidence intervals. The mean age and proportion of females were calculated for the subsequent years along with 95% confidence intervals. These were compared to determine whether these variables had changed over the years.

The trend in incidence of CRPS in general was derived from the hospital-based data by using the absolute numbers of newly opened DBC codes for CRPS as well as relative frequencies. The mean age and gender of patients suspected of having CRPS were compared between the years using the same method as described for CRPS after DRF.

The incidence rate of suspected CRPS in general in the Netherlands was calculated by dividing the number of newly opened DBC codes for CRPS, as extracted from national DIS data (numerator), by the Dutch mid-year population (denominator). Subsequently, the trend in the incidence of CRPS was assessed by describing the absolute number of newly opened DBC codes each year and by calculating the annual incidence rates of suspected CRPS along with their 95% confidence intervals. Additionally, to determine whether there was a significant trend in incidence we performed a Poisson regression analysis, the population was added as the offset value. To allow comparison with the incidence rate calculated by De Mos et al., the standard morbidity ratio (SMR) was calculated using the indirect standardization method,^25^ standardizing according to age and gender, and using the study population of De Mos et al. as the standard population.^1^

## Results

### Hospital-based trend in incidence of CRPS after DRF

A total of 5488 patients with a DRF were included. There were 30 suspected cases of CRPS, and of these, 20 were confirmed after reviewing the medical records. The other 10 cases did not meet the Budapest criteria. The proportion of suspected cases that were confirmed after review of medical records was 0.67 (95% CI: 0.47–0.83).

Descriptive characteristics are displayed in Table 1. We found an incidence of CRPS after DRF of 0.36%. The difference in the incidence of CRPS after DRF between the three centres is shown in Table 2.

**Table 1.** Age and sex of patients after DRF without CRPS, suspected of having CRPS and with confirmed CRPS.

	No CRPS	Suspected CRPS	Confirmed CRPS	*p* value^a^
Cases	5458	30	20	
Mean age (SD)	42.7 (27.6)	53.8 (15.9)	53.6 (15.2)	0.04
Female (%)	3322 (61)	24 (80)	15 (75)	0.1

CRPS: complex regional pain syndrome; SD: standard deviation.

a Comparing the group with confirmed CRPS to the group with no CRPS.

**Table 2.** Incidence of CRPS in the three participating centres.

Centre	Incidence ratio (%)	*p* value^a^
1	0.09	
2	0.08	0.87
3	0.93	0.06

a *p* value compared to centre 1.

There was no significant difference in the annual incidence rates of CRPS after DRF (see Table 3). For patients with CRPS, there was no significant difference over the years in mean age (2014: 70 years, based on 2 patients, therefore the 95% CI was not calculated; 2018 52.6 years, 95% CI: 31.0–74.2) or proportion of females (2014: 0.5, 95% CI: 0.01–0.99; 2018: 0.6, 95% CI: 0.15–0.95). In addition, for patients suspected of having CRPS, there was no significant difference over the years in mean age (2014: 61.9 years, 95% CI: 54.4–69.4; 2018: 52.6 years, 95% CI: 31.0–74.2) or proportion of females (2014: 0.8, 95% CI: 0.44–0.97; 2018: 0.6, 95% CI: 0.15–0.95).

**Table 3.** Annual incidence rates of CRPS after DRF.

Year	DRF cases	CRPS cases	Incidence (%)	95% CI
2014	827	2	0.24	0.07–0.88
2015	1062	5	0.47	0.20–1.10
2016	1210	8	0.66	0.34–1.30
2017	1181	0	0	–
2018	1208	5	0.41	0.18–0.97

DRF: distal radius fracture; CRPS: complex regional pain syndrome; CI: confidence interval.

### Hospital-based trend in the incidence of CRPS in general in the Netherlands

**Figure 3.**
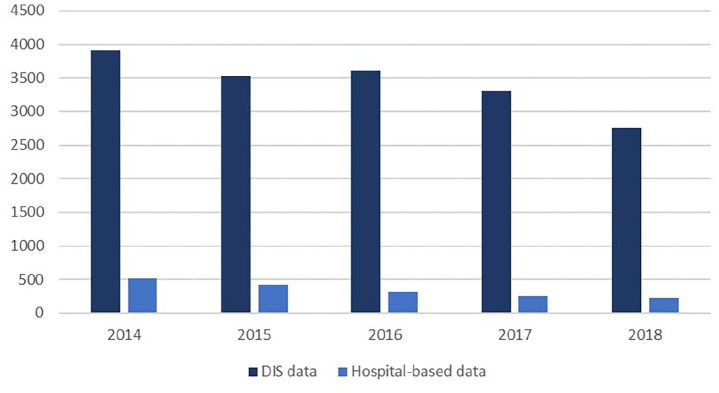
The chart shows the trends in opened DBC codes for CRPS based on DIS data and hospital-based data.

Between 2014 and 2018, a total of 1737 new DBC codes for CRPS were opened in the participating centres. The annual numbers of newly opened DBC codes for CRPS are displayed in Figure 3. The centres included in this study comprised 10% of all the DBC codes for CRPS in the Netherlands between 2014 and 2018.

The mean age of all suspected CRPS cases was 48.8 years (95% CI: 48.1–49.6). There was no significant difference between the age of males and females (males: 49.7; 95% CI: 48.2–51.3; females: 48.6; 95% CI: 47.7–49.5). The mean age remained similar during the study period: 48.5 years in 2014 (95% CI: 47.1–49.9) and 47.6 years in 2018 (95% CI: 45.4–49.8). The distribution of age is shown in Supplement A (Supplemental material). The proportion of females was 0.78 (95% CI: 0.76–0.80), ranging from 0.75 in 2014 (95% CI: 0.71–0.79) to 0.78 in 2018 (95% CI: 0.72–0.83).

### Population-based trend in the incidence of CRPS in general in the Netherlands

A total of 17,114 new DBC codes for CRPS were opened in the Netherlands from 2014 to 2018. The annual numbers of newly opened DBC codes for CRPS are displayed in Table 4.

**Table 4.** Crude and standardized incidence rates of CRPS according to calendar year.

Year	Mid-year population^a^	Cases	Crude IR	Standardized IR^b^	SMR^b^
2014	16,865,007.5	3909	23.2 (22.5–23.9)	20.9 (20.2–21.5)	0.71 (0.69–0.73)
2015	16,939,923	3526	20.8 (20.1–21.5)	18.6 (18.0–19.2)	0.63 (0.61–0.65)
2016	17,030,313.5	3608	21.2 (20.5–21.9)	18.8 (18.2–19.4)	0.64 (0.62–0.66)
2017	17,131,295.5	3305	19.3 (18.7–20.0)	17.0 (16.4–17.6)	0.58 (0.56–0.60)
2018	17,231,623.5	2766	16.1 (15.5–16.7)	14.1 (13.6–14.6)	0.48 (0.46–0.50)
Total	85,198,163	17114	20.1 (19.8–20.4)	17.8 (17.6–18.1)	0.61 (0.60–0.61)

IR: incidence rate.

a Using population numbers of the Central Bureau of Statistics (CBS).

b Using the study population of De Mos et al. as the standard population.

**Figure 4.**
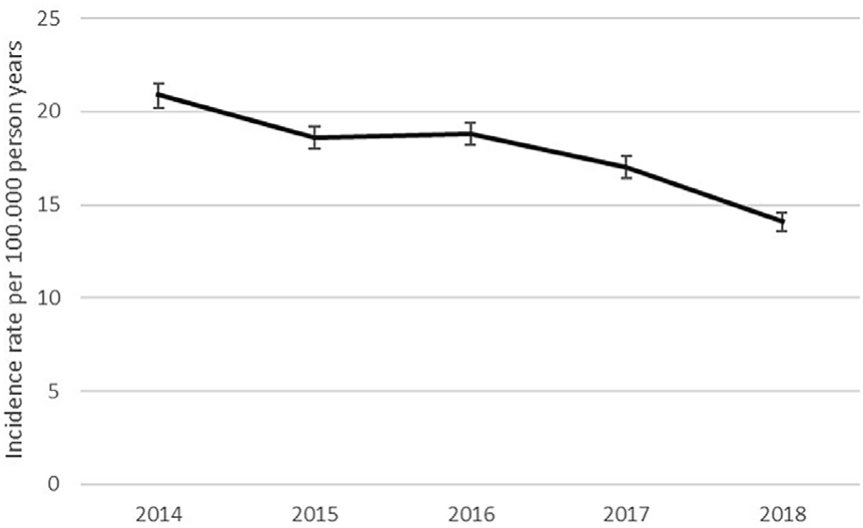
The graph shows the crude annual incidence rates of CRPS in the Netherlands with upper and lower 95% confidence intervals.

The incidence rate of suspected CRPS over the whole study period was 20.1 (95% CI: 19.8–20.4) per 100,000 person years. The trend in the annual incidence rates of suspected CRPS is illustrated in Figure 4. The annual incidence decreased from 23.2 (95% CI: 22.5–23.9) per 100,000 person years in 2014 to 16.1 (95% CI: 15.5–16.7) per 100,000 person years in 2018. The Poisson regression analysis showed a significant trend with a yearly decrease in incidence of 7.6% (*p* < 0.001). The SMR for the whole study period was 0.61 (95% CI: 0.60–0.61), meaning that we found an incidence rate of cases suspected for CRPS that was 0.61 times the incidence rate of confirmed CRPS cases found in the study of De Mos et al.^1^ In other words, we found a 1.6 times lower incidence rate. After standardizing the annual incidence rates to the study population of De Mos et al., the decreasing trend in incidence was sustained: 20.9 (95% CI: 20.2–21.5) per 100,000 person years in 2014 to 14.1 (95% CI: 13.6–14.6) per 100,000 person years in 2018 (see Table 4).

## Discussion

The reported incidence of CRPS varies widely between 5.5 per 100,000 person-years in the United States and 26.2 per 100,000 person-years in the Netherlands.^1,2^ CRPS, especially after a distal radius fracture (DRF), is frequently studied and has a reported incidence between 0.8% and 37%.^5–7,26^ The current study provides data on the epidemiology of CRPS after a DRF and CRPS in general.

This study found that the incidence of CRPS after a DRF was 0.36%. This incidence is among the lowest incidences reported in literature, which vary between 0.8% and 36%.^5–7,26^ There was no trend in the annual incidence rates of CRPS after DRF. However, proving a significant decrease in incidence of CRPS after DRF is very hard since the incidence is near 0% and therefore, it would require a very large study population. Regarding CRPS in general, the hospital-based data showed a decrease in absolute number of cases suspected for CRPS. This is supported by the national data, which shows that the incidence of CRPS gradually decreased from 2014 to 2018 from 23.3 to 16.1 per 100,000 person years. Over the whole study period, an incidence rate of 20.3 per 100,000 person years was found, which was 1.6 times lower than the incidence rate found by De Mos et al. in 2007.

The changing approach towards CRPS, as described in recent literature, is a plausible explanation for the low incidence of CRPS after DRF and decreasing trend of incidence of CRPS in general found in this study.^2,9,12^ Over the last decade, a different perspective on the etiology of CRPS has been raised. There are suggestions that we should stop stigmatizing CRPS as a severe debilitating disease and rather describe it as a variation of the healing process.^27,28^ This view is supported by recent insights into the importance of the length of cast immobilization and prevention of disuse in the development of CRPS after a DRF.^14–16^ For example, Terkelsen et al. showed that 4 weeks of forearm immobilization causes symptoms very similar to those associated with CRPS. Furthermore, similarities are noted between CRPS, other pain syndromes, and functional neurological disorders (FND), suggesting new points of engagement for prevention and therapy.^13,27,29^ Linton and Shaw described how catastrophic thinking, an exaggerated negative orientation towards pain, leads to fear and disuse and is a marker of the development of long-term problems in patients with pain syndromes such as low back pain. This is consistent with findings by Teunis et al.,^27^ who found that catastrophic thinking plays an important role in developing and maintaining symptoms of CRPS.

Based on these findings, a shift is seen in clinical practice and in recent research towards shorter cast immobilization for non-operative treated fractures of the distal radius. This study found an incidence of CRPS of 0.36% after a DRF. This low incidence in this study might be explained by the shift in treatment with shorter immobilization periods for DRF in the Netherlands. Shorter periods of immobilization might lead to less disuse and posttraumatic pain. A recent systematic review of Delft et al.^30^ showed that for non-reduced DRF shorter cast immobilization leads to better or similar patient reported outcomes. In addition to the length of immobilization, informing the patient properly, managing the patient’s expectations and encouraging normal use of the limb, with for example an active home exercise programme, is evenly so important to manage the psychological impact of a trauma and thereby prevent disuse and disproportionate pain.^28^ An active home exercise programme is often based on the same principles as PEPT treatment, namely the assumption that behavioural and psychological factors can exacerbate pain and dysfunction.^23,31^ Further research should be done to investigate whether short immobilization periods including home exercise programmes for DRF, is safe and leads to less posttraumatic pain and better patient reported outcomes.

Another explanation for the decreasing incidence of CRPS found in this study is having more scrutiny in the assessment of the differential diagnosis of CRPS. The diagnostic criteria state that ‘CRPS is excluded if another diagnosis can better explain the signs and symptoms’. This is best insured by a specialized multidisciplinary team, as recently stated by a task force of the European Pain Federation.^32^ It has been shown that, in the majority of the cases, these teams can make an alternative diagnosis through assessment of patients suspected of CRPS.^33^ In the Netherlands, there are six centres with such expert-teams in CRPS, of which three were included in this study. Differences in interpretation of ‘continuous and disproportionate pain’ can influence the incidence of CRPS as well. In our study, a difference was found between the incidence of CRPS after DRF in one centre and that in the other two centres, most likely due to differences in definition. Previous studies have mentioned this as a possible influence on the incidence as well.^1,2,34^

There are a number of limitations to this study, which were mainly inherent to the use of a retrospective administrative database. Regarding the incidence rate of CRPS, a possible weakness was the absence of a specific DBC for CRPS within rehabilitation medicine. As a result, we were unable to include cases that might have been diagnosed by a physiatrist, possibly resulting in an underestimation of the incidence rate of CRPS. Second, the use of only hospital-based data in this study might explain the low incidence rate found in this study, especially in comparison with the incidence rate found by De Mos et al. which was based on a primary care population. In their study, only 75% of the cases were confirmed by a specialist. However, the general opinion is that CRPS should be diagnosed by an expert; therefore, hospital-based data should represent the incidence of CRPS more accurately. Moreover, we expect that any degree of underestimation is exceeded by the degree of overestimation due to the large proportion of suspected cases of CRPS included and the possibility that the same patient could be registered multiple times under different DBC codes for CRPS belonging to different specialties. In addition, we expect that the degree of under- or overestimation is continuous over the years and thus does not influence the trend in the incidence of CRPS. Third, a limitation of the hospital-based data was that it only comprised data from three of the six trauma centres in the Netherlands with expertise in CRPS. Unfortunately, three centres declined participation in this study. However, the data from the three participating centres were so evident that we do not think we would have found a much different trend in incidence, using the data of all six hospitals. Finally, the reliability of the diagnosis of CRPS in itself is a limitation. The criteria have been changing over the years and remain subjective as shown by the difference in diagnosis of CRPS between hospitals.^6,9,35^ As pointed out by Ring et al.,^4^ pain is a continuous scale rather than a dichotomous outcome and correlates better with the psychological aspects as pointed out earlier than with measures of pathophysiology or impairment. This remains a limitation of all studies regarding CRPS and makes it difficult if not impossible to interpret and compare results of previous incidence studies. However, during the study period of this study, these criteria have remained unchanged and there has been no change in policy regarding CRPS of the different hospitals. Therefore, we expect that its influence on the incidence is the same over the years and does not influence the trend in incidence of CRPS.

## Conclusion

This study shows an incidence of CRPS after DRF which is among the lowest incidences reported in literature. The incidence of CRPS in general seems to decrease over the years. The changing clinical approach towards CRPS, with more focus on prevention and the psychological aspects of posttraumatic pain may explain this decrease in CRPS incidence. Future practice and research should focus on prevention and treatment of posttraumatic pain.

## Supplemental Material

sj-jpg-1-bjp-10.1177_20494637211041935 – Supplemental material for Decreasing incidence of complex regional pain syndrome in the Netherlands: a retrospective multicenter study Click here for additional data file. Supplemental material, sj-jpg-1-bjp-10.1177_20494637211041935 for Decreasing incidence of complex regional pain syndrome in the Netherlands: a retrospective multicenter study by Tjitske D Groenveld, Emily Z Boersma, Taco J Blokhuis, Frank W Bloemers and Jan Paul M Frölke in British Journal of Pain
