# Supplementary figures and images for: Decreasing incidence of complex regional pain syndrome in the Netherlands: a retrospective multicenter study
Source: Br J Pain. 2021 Sep 6;16(2):214–22. doi: 10.1177/20494637211041935 (PMC8998521; doi:10.1177/20494637211041935)

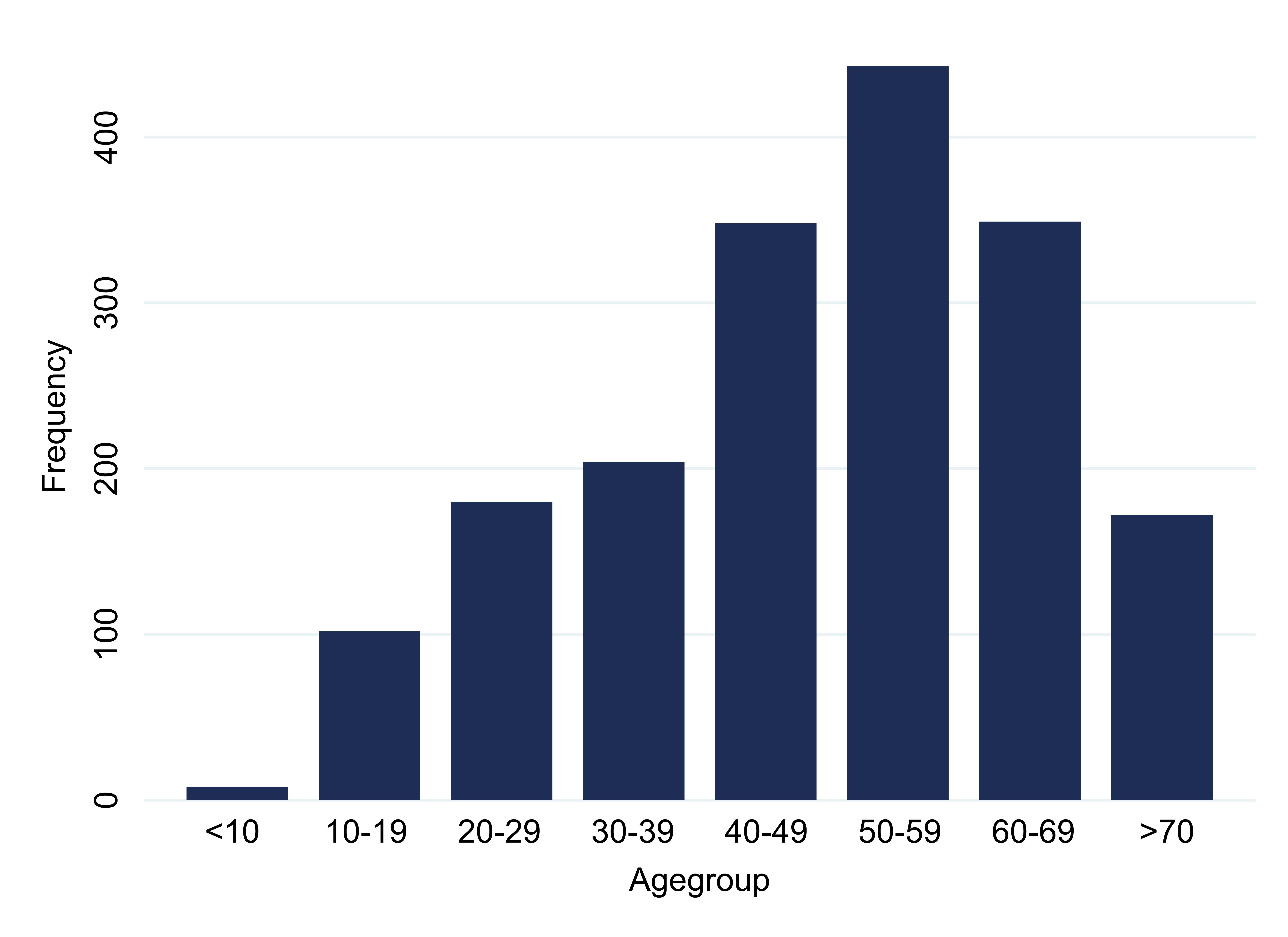

Supplement: sj-jpg-1-bjp-10.1177_20494637211041935 – Supplemental material for Decreasing incidence of complex regional pain syndrome in the Netherlands: a retrospective multicenter study [file sj-jpg-1-bjp-10.1177_20494637211041935.jpg]
